# Eating Patterns and Food Choices of Latvian Infants during Their First Year of Life

**DOI:** 10.3390/medicina54010007

**Published:** 2018-03-23

**Authors:** Inga Sirina, Ieva Strele, Inese Siksna, Dace Gardovska

**Affiliations:** 1 Department of Pediatrics, Doctoral Department Faculty of “Medicine”, Riga Stradiņš University, Riga LV 1007, Latvia; inese.siksna@ldusa.lv; 2 Institute of Food Safety, Animal Health and Environment BIOR, Riga LV 1076, Latvia; 3 Department of Public Health and Epidemiology, Riga Stradiņš University, Riga LV 1010, Latvia; ieva.strele@rsu.lv; 4 Department of Paediatrics, Children’s Clinical University Hospital, Riga Stradiņš University, Riga LV 1004, Latvia; dace.gardovska@rsu.lv

**Keywords:** breastfeeding, food choices, infants, nutrition, toddlers

## Abstract

*Introduction*: Pregnancy, infancy, and early childhood are periods of rapid growth and development. The role of nutrition is very important during these critical growth and development periods. The aim of the study was to investigate infant feeding practices through the first year of life in Latvia, and to compare feeding practices with nutritional guidelines in Latvia and other European countries. *Methods*: We analysed cross-sectional study data from food frequency questionnaires with additional questions on breastfeeding and complementary feeding introduction. A total of 266 infants from all Latvian regions from birth to 1-year-old were included in the study. Breastfeeding rates were assessed by month of age. Complementary feeding was assessed using three age groups (0–3.9 months, 4–6.9 months, and 7–12.9 months), whereas two groups were used to assess food frequency and portion sizes (0–5.9 months and 6–12.9 months). *Results*: The breastfeeding rate during the first month of life was 89%. At 6 months, 68% of infants received breast milk, but by 12 months, only 45% still received breast milk. Complementary foods were introduced at a mean age of 5 months (standard deviation = 1). Before 4 months of age, 9% of infants were receiving complementary food, the majority (85%) between 4 and 6 months of age. There were 6% of infants who were introduced to complementary foods after 7 months of age. First complementary food choices were mainly porridge (64%), vegetables (21%), and fruits (10%). After 6 months of age, foods from almost all food groups were present in each infant diet at least once per day, such as vegetables (85%), potatoes (85%), fruits (81%), dairy (78%), and meat (73%), less than once per day—grains (88%), fats (73%), cow’s milk (66%), eggs (45%), fish (36%), and legumes (28%). *Conclusion*: Breastfeeding rate during first months of life is high in Latvia. Breastfeeding was sustained at the age of 6 months, in the highest rate among Baltic countries. However, only 45% continued breastfeeding at the age of 12 months, in accordance with WHO recommendations. A guideline on complementary feeding is followed by the majority of parents. There are deviations from guidelines in inclusion of some foods in the diet and frequency of consumption.

## 1. Introduction

Pregnancy, infancy, and early childhood are periods of rapid growth and development. The role of nutrition is very important during these critical growth and development periods. Inappropriate nutrition places infants at risk of impaired emotional and cognitive development and adverse health outcomes [1].

The World Health Organization (WHO) recommends exclusive breastfeeding during the first six months of life. From six months onwards, continued breastfeeding combined with complementary foods of good quality in sufficient quantities for 2 years or longer is recommended. The WHO recommendation applies to all countries and populations regardless of economic status or developmental level [2]. Latvian recommendations on breastfeeding were published by Ministry of Health in year 2003, and are in line with WHO recommendations [3]. The latest data on breastfeeding duration amongst Latvian infants is available from the year 2008. It was published as part of a longitudinal study by The Institute of Anatomy and Anthropology, Riga Stradiņš University [4].

Feeding practices, such as breastfeeding and the timing of introduction of complimentary foods, can influence the risk of infants being overweight and obese in adolescence age [5]. Poor dietary intake may trigger metabolic programming pathways which predispose infants to chronic diseases in later life. Furthermore, exposure to different foods in early life shapes the development of food preferences and eating behaviours in later life [6]. Together, these factors emphasise the importance of focusing on nutrition practices during early life to protect future health.

At present, there are no Latvian national data reflecting nutritional habits during the first year of life. This research will give an overview of Latvian infants’ eating habits. The data can inform healthcare professionals about infant’s nutritional habits and their deviations from recommendations, in order to assist parents in improving their infant’s diets.

## 2. Materials and Methods

In 2012, with the support of the European Food Safety Authority (EFSA) The Institute of Food Safety, Animal Health and Environment BIOR started a cross-sectional study which included 3420 participants covering different age groups from birth to 74 years [7]. Data collection for infants and toddlers was started in 2012 and was completed in 2014. Target sample size was 560 infants and toddlers (0–36 months). Two-stage sampling was used to select infants and toddlers. In the first stage, practices of family doctors and paediatricians from all Latvian regions were selected. In Latvia, every infant is registered at family doctor or paediatrician right after birth. In the second stage, doctors selected infants and toddlers from their patients register.

According to the survey methodology report, selection of practices was representative for the target population, whereas an exact procedure of selection of respondents from each doctor’s register is less clear. Still, it is stated that the presence of selection bias is unlikely [7]. Besides, the characteristics of our study population are in line with Latvian population demographics (Table 1). If parents agreed to participate in the study, interviewers contacted them and collected nutritional data. The interviewers had to have an appropriate education with medical background, as well as experience in nutritional data collection in similar studies. Interviewers had training before starting the survey. Guidelines were given to each interviewer with all necessary information for data collection.

Parents or primary caregivers of the infants and toddlers completed a food frequency questionnaire (FFQ) and a 24 h dietary recall about their infant’s diet. FFQ included additional questions on breastfeeding and complementary feeding introduction. Paper versions of both questionnaires were used. The following data were collected: general information about characteristics of the parents and child, feeding practices—breastfeeding, formula feeding, complementary feeding, consumption of food products from all food groups, beverages, eating habits, and possible food safety risks were observed.

For estimating portion sizes, a validated food quantification picture book in print and electronic version and a liquid measuring cup were used. Interviews were conducted in Latvian and Russian.

Breastfeeding and complementary feeding definitions were strictly defined in accordance with the WHO criteria [8]. Complementary feeding in this paper is defined as introduction of any food besides human milk or infant formula. Several nutritional guidelines were used (WHO, EFSA, Finnish national recommendations, German national recommendations) to evaluate breastfeeding, food choices, and complementary foods introduction timing [9,10,11,12].

The analyses presented in this paper focus on 270 infants from birth to 1-year-old (0 to 12 months). Four participants were excluded because they were missing all or part of the necessary information on feeding patterns. Therefore, the final number of analysed participants was 266 infants. In the present data analysis, FFQ data were used. The two 24-h dietary recalls were used only for nutritional data validation. Breastfeeding and complementary feeding introduction data were from additional questions in the FFQ.

Data were collected in an Excel spreadsheet and analysed with the IBM SPSS Statistics 22 programme. Nutritional data were analysed using the national food composition database. As frequency of consumption of complementary foods and a portion size were not normally distributed (Shapiro–Wilk test), they were summarized as median values with interquartile range (IQR). Age of infants and age of mothers were summarized as mean values with standard deviation (SD). The Pearson Chi-squared test was used to compare proportions. A *p*-value less than 0.05 was chosen to indicate statistically significant difference. 

The study was approved by Riga Stradiņš University Ethics committee on 12 September 2013.

## 3. Results

A general description of the study participants is available in Table 1. Overall, proportions of boys and girls, as well as regional distribution of study participants were comparable with corresponding demographic characteristics of Latvian population. The mean age of mothers enrolled in the study was 29 years (SD, 6) and the mean age of mothers in 2012 according to the Latvian statistics was 29 years.

### 3.1. Breastfeeding

**Figure 1:**
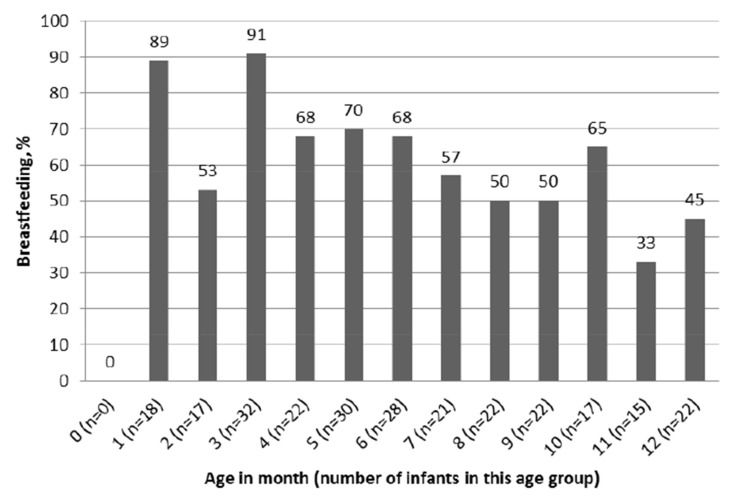
Proportion of breastfed infants within the age group.

Breastfeeding prevalence was assessed by age in one-month intervals (Figure 1). Almost all infants (89%) were breastfed during the first year of life. From 6 months of age onwards, breastfeeding prevalence was below 70%. The breastfeeding rate decreased with the infants’ age (*p* = 0.002). The lowest breastfeeding rate was observed during 11 and 12 months, 33% and 45%, accordingly.

### 3.2. Complementary Feeding

A total of 164 of the infants in our sample had started complementary feeding. The mean age of complementary feeding initiation was 5 months (SD = 1). Before 4 months of age, 9% (*n* = 14) of infants were receiving complementary food. The majority of parents (85%) introduced complementary foods to their infants 4–6 months of age. There were 6% (*n* = 10) of infants who were introduced to complementary foods after 7 months of age. The most frequent food choice at first weaning was porridge (64%); the second most frequent food choice was vegetables (21%). The most popular vegetables were pumpkin, potatoes, and squash. In 10% of cases, weaning was initiated by fruit puree and in 5% with other foodstuffs (for example, yoghurt).

### 3.3. Food Choices

Before 4 months of age, only a few infants consumed any complementary food. More food variety was introduced in 4–6.9-month olds, including vegetables, potatoes, fruits and berries, as well as grains. After 7 months of age, foods from almost all food groups were present in each infant’s diet, such as grains, vegetables and fruits, meat and fish, eggs, legumes, dairy products, and others (Table 2).

For further analysis, all infants were divided in two groups: 0–5.9 months and 6–12.9 months. More subgroups were not reasonable due to the small sample size in younger age groups.

Vegetables were consumed by 86% (*n* = 126) of infants after 6 months of age (*n* = 126). Median consumption frequency was once a day, and median one portion size was 80 g (Table 3). Before 6 months of age, vegetables were consumed by 10% (*n* = 12) of infants.

Potatoes were analysed separately from the vegetable group. Potatoes were consumed by 85% (*n* = 124) of infants aged over 6 months in a median frequency of once a day. The median amount consumed during one feeding time was 17 g. Before 6 months of age, potatoes were consumed by 8% (*n* = 9) of infants. 

All food groups of complementary feeding were mainly consumed after the age of six months. Among infants older than six months, 81% (*n* = 118) consumed fruits and berries. The median frequency of consumption was once per day, and the median amount was 54 g in one feeding. Consumption of grains was also common (88%), but the median frequency was less than once a day (6 times per week) and the median amount of grains consumed in one feeding was 20 g. Meat was also introduced in a diet of majority (73%, *n* = 107) infants. The median frequency of meat consumption was 1.7 times a day, and median size of portion was 46 g in one feeding. About one third (36%, *n* = 53) of infants in this age group consumed fish. Consumption of fish was relatively rare; the median frequency of eating fish was once a week, and the median amount of fish in one feeding was 30 g.

Consumption of both milk and dairy products was quite common among infants older than six months of age. Milk and dairy products were introduced in the diets of 66% (*n* = 96) and 78% (*n* = 114) of infants, respectively. The median consumption frequency was less than once a day for milk and once a day for dairy products; the median amount of product per one feeding was 45 mL or 46 g of milk, and 45 g of dairy products.

Almost half (45%, *n* = 65) of infants after the age of six months had eggs in their diet; the median frequency and amount of consumed eggs was 1 egg per week. Less common was consumption of legumes. They were introduced in the diets of 28% (*n* = 41) infants in this age group. The frequency of consumption was only once a month, and the median amount per one feeding was 30 g.

After the age of six months, the majority (73%, *n* = 107) of infants consumed also fats. The median frequency of fat consumption was 5 times a week, and the median amount was 6 g during one feeding.

Among infants younger than six months, only 6% (*n* = 7) consumed fruits and berries, and 5% (*n* = 6) consumed grains. A few infants had meat and fish introduced in their diets (3%, *n* = 3 and 1%, *n* = 1, respectively). Either milk or dairy products were introduced in the diet of 3% (*n* = 3) infants below six months of age. Only 1% (*n* = 1) of infants in this age group consumed legumes and 3% (*n* = 4) consumed fats.

No statistically significant differences were found in different food group consumption and mother’s education (Supplementary Material, Table S1; Table S2).

## 4. Discussion

This survey investigated feeding practices, such as breastfeeding, complementary feeding and food choices, of infants during the first year of life in Latvia.

It is worth mentioning that it is difficult to compare rates of breastfeeding and feeding practices between different recommendations, but even more, it is difficult to compare different studies, owing to different definitions, methods of sampling, and data collection.

According to the WHO (World Health Organisation) and Latvian recommendations, exclusive breastfeeding should continue for the first 6 months, and breastfeeding with complementary foods until 24 months [3,9]. Results of the present survey estimated that the current rate of starting breastfeeding during first month of life in Latvia is 89%. Comparing this research data with Nordic countries, breastfeeding duration in Latvia is among the highest national breastfeeding rates at the age of 6 months, rates being: Finland (58%), Sweden (63%), Latvia (68%), Iceland (74%), and Norway (80%) [13]. The Latvian breastfeeding rate at 6 months is the highest in the Baltic states—rates for the others were Lithuania (31%) and Estonia (40%) [13]. Compared to the latest published data on breastfeeding duration in Latvia, there is an increase of breastfeeding duration. Breastfeeding rate from birth decreased from 94% to 89%, at 6 months of age, increased from 58% to 63%, and at 12 months, increased from 39% to 45% [4].

Recommendations on the time when complementary foods should be first introduced, differs. Latvian national nutritional recommendations are aligned with WHO guidelines, and recommend starting complementary feeding at 6 months of age with small amounts of food, and increasing the quantity as the child gets older, while maintaining frequent breastfeeding [3,9], whereas the EFSA clearly state that complementary foods have to be introduced into the diet of infants between 4 and 6 months [10]. The same timing is given by Finnish feeding guidelines. According to these recommendations, solid foods can be introduced to the child in tasting portions at the age of 4–6 months at the earliest. All children need solid food from the age of 6 months onwards. For children who do not get breast milk at all, solid food should be started at 4–6 months of age. German recommendations on infant nutrition declare that most children can propel boluses of semisolid food with their tongues from the age of 4 to 5 months onward [12]. Similar findings on complementary food introduction were found in Italian research, where, at 4 and 6 months of age, 34.2% and 85.5% of infants, respectively, had been introduced to semi-solid food [14].

Study results estimated that the introduction of complementary foods is started mainly between 4 and 6 months (85%). Only a few infants were introduced to complementary foods too early (9%) or too late—after 7 months (6%).

Contrary to precise guidelines on nutrient intake during the first year of life, there are no determined and aligned guidelines on different food product introduction timings in a diet, frequency, and amounts of consumption; cows’ milk is an exception. Cow’s milk is not recommended to be introduced before one year of age [15,16,17]. However, ESPGHAN notes that small quantities of cow’s milk can be given as part of food and milk alternatives and can be introduced as complementary foods after the introduction of iron-rich foods. Early cow’s milk introduction and consumption of more than 500 mL is related with iron deficiency [18]. According to these research results, consumption of cow’s milk is quite limited. Cow’s milk before one year of age is consumed by 66% of infants, but it is consumed less than once a day and in the amount of 45 mL per feeding. Fermented milk products are introduced mainly after 6 months of age, (78% of infants). According to Latvian nutritional recommendations for infants, fermented milk products have to be introduced only after one year of life [3]. Finnish recommendations declare that fermented milk products can be introduced starting at 10 months [11]. There are no clear indications on fermented milk product consumption in other recommendations.

Meat is emphasised as an important source of heme iron that is highly bioavailable compared with non-heme iron sources found in meat alternatives (e.g., legumes) and iron-fortified cereals [19]. The data on meat consumption in our study is satisfactory. At the age of 6 months and older, meat is introduced by 73% in significant amounts per feeding, and of a frequency of almost twice a day (median = 1.7). In 2003, the WHO and the Pan American Health Organization published unified, scientifically based guiding principles for complementary feeding of the breast-fed child. The guidelines recommend that “meat, poultry, fish, or eggs should be eaten daily, or as often as possible,” to ensure that the nutrient needs of the child are met [20]. Canadian recommendations for healthy term infants from 6 to 24 months recommend iron-rich complementary foods, such as meat and meat alternatives 2 or more times a day [19]. The EFSA and Finnish recommendations state that a mixture of vegetables, potato, and meat can be given as the infant’s first semisolid food (with oily fish instead of meat once or twice a week) to provide highly bio-available iron and zinc [10,11]. German guidelines declare that the early consumption of meat, liver, and fish is associated with thriving growth and good cognitive development later on in childhood [12]. According to Latvian recommendations, meat has to be introduced at 8 months of age [3]. Despite the fact that fish is highly recommended to be consumed, replacing meat once or twice a week, research data shows that fish is consumed only by 36% after 6 months of age, once a week.

According to Latvian recommendations, eggs have to be consumed only after one year of age. In this research, it was found that eggs are consumed after 6 months of age, by 45% in a frequency of 1 egg per week. Contrary to Latvian recommendations, the WHO and Finnish nutritional guidelines allow egg consumption before one year of age [9,11].

There is alignment between guidelines that vegetables have to be introduced as one of the first complementary foods. According to research data, vegetables and potatoes are very popular amongst food choices, and are introduced after 6 months of age, by 86% and 85%, respectively, and before 6 months of age, by 10% and 8%, respectively. Similar to vegetables, fruits and berries are consumed on a regular basis by 81% of infants aged over 6 months. According to Latvian recommendations, fruits and berries can be introduced at the same time as vegetables [3]. Such recommendations are supported by Finnish nutritional guidelines [11]. 

The WHO, Finnish, and Latvian nutritional guidelines state that porridge can be chosen as one of the first complementary foods [3,9,11]. The EFSA and German nutritional guidelines recommend introducing porridge after the first complementary foods [10,12]. In this study, it was found that cereals are consumed mainly after 6 months of age by 88% of infants. Unfortunately, the majority of guidelines do not describe how much and how frequently cereals have to be consumed. Only the EFSA mentions that 20 g of cereals are satisfactory for one portion [10]. Unfortunately, these guidelines do not describe the frequency of consumption. This study shows that cereals are consumed less than once a day in the amount of 20 g during one feeding. This could be unsatisfactory.

Legume consumption is not described in the reviewed recommendations, except Latvian nutritional guidelines, which state that legumes can be introduced at 8 months of age [3]. In this study, it was found that only 28% of infants aged 6 months and over consume legumes and on a very rare basis in small amounts.

It is clear that a variety of nutrient-rich foods should be fed to ensure that all nutrient needs are met. Comparison with several nutritional guidelines helps to assess feeding practices, but detailed assessment of nutrient intakes is still needed. Overall nutritional guidelines on complementary feeding and food choices are followed by the majority of infants. The concern is about breastfeeding duration. Actions within public health initiatives have to be done to protect and promote breastfeeding at least during the first year of life.

There were several limitations and challenges during the study. When it comes to nutritional data assessment, there is always a possibility of the parent or caregiver over- or underreporting an infant’s daily food or drink intake. Parents did not receive any significant incentives (only measuring cup and food pyramid sticker) for participation in the study. At the same time, involvement in terms of time from parent’s side had to be quite high. It took approximately 1.5–2 h to collect all necessary data for FFQ and 24 h dietary recall questionnaires. It could lead to additional mistakes in reporting infant’s daily diet. A major strength of a study is professional interviewers who have collected nutritional data. Other strengths worth mentioning are validated questionnaires and The Institute of Food Safety, Animal Health and Environment BIOR nutritional database, which allows the study to be repeated quite precisely.

## 5. Conclusions

In conclusion, this study provides information on breastfeeding duration, complementary food introduction, and food choices from different product groups. After research data comparison with several guidelines, the conclusion is that breastfeeding duration during the first year of life is not long enough, and initiatives should be done to increase awareness about breastfeeding benefits and support possibilities for breastfeeding mothers. Complementary feeding initiation is done in the appropriate time for the majority of observed infants, and there is little concern about introduction being too early or too late. It is difficult to reach a conclusion on appropriate food choices due to a lack of precise guidelines. Overall, there is a variety of food consumed by infants. This study data on eating pattern and food choices, as well as their comparison with different guidelines, can be used to inform health care professionals within public health initiatives. The data can be used to improve the diets of infants and toddlers, and to guide parents regarding breastfeeding and appropriate food selection during the first year of life. Furthermore, this information is useful for monitoring changes in the eating habits of Latvian infants and toddlers over time.

## Figures and Tables

**Table 1:** General description of study population in comparison with Latvian demography statistics 2012.

Parameter		Study Group	Latvian Statistics on New-Borns and Mothers, 2012
New-borns, *n*		**266**	19,897
Infant gender, *n* (%)	Girls	126 (47)	9640 (48)
	Boys	140 (53)	10,257 (52)
Infant residence, *n* (%)	Riga	88 (33)	6739 (34)
	Riga region	36 (13)	3982 (20)
	Other cities	43 (16)	3171 (16)
	Countryside	99 (37)	6005 (30)
Mother’s age, *n* (%)	17–25 years	71 (27)	6414 (32)
	26–35 years	156 (58)	10,946 (55)
	36–45 years	39 (15)	2418 (12)
Mother’s education, *n* (%)	University degree	149 (56)	Not available
	Secondary education	93 (34)	Not available
	Primary education	19 (7)	Not available
	None	3 (1)	Not available
	No information	2 (1)	Not available
Feeding choice, *n* (%)	Breastfeeding	168 (63)	Not applicable
	Infant formula feeding	98 (37)	Not applicable
	Complementary feeding introduced	164 (65)	Not applicable

**Table 2:** Infants consuming product group within the age group.

**Product Group**	**Age in Months**		
	0–3.9	4–6.9	7–12.9
	(*n* = 69)	(*n* = 79)	(*n* = 118)
	*n* (%)	*n* (%)	*n* (%)
Vegetables	3 (4)	22 (28)	112 (95)
Potatoes	1 (1)	22 (28)	110 (93)
Fruits and berries	1 (1)	22 (28)	102 (86)
Grains	4 (6)	17 (22)	113 (96)
Meat	2 (3)	8 (10)	100 (85)
Fish	1 (1)	3 (4)	50 (42)
Milk	1 (1)	12 (15)	86 (73)
Dairy	1 (1)	8 (10)	108 (92)
Eggs	0 (0)	4 (5)	61 (52)
Legumes	1 (1)	2 (3)	39 (33)
Fats	1 (1)	12 (15)	98 (83)

**Table 3:** Median frequency of consumption and median consumed amount per feeding by food groups among infants aged from 6 to 12.9 months.

Product Group	Feeding Times		Portion Size	
	Per Day or Week	IQR *	Per Feeding in Grams	IQR*
Vegetables	1/day	0.43–1	80	50–152
Potatoes	1/day	0.43–1	17	10–50
Fruits and berries	1/day	0.43–1	54	40–100
Grains	6/week	3–14	20	10–38
Meat	1.7/day	1.5–3.2	46	20–58
Fish	1/week	0.5–1.6	30	12–50
Milk	6/week	3–10	46	19–108
Dairy	1/day	1–2	45	30–57
Eggs	1/week	0.25–1.7	1 egg	1–1
Legumes	0.25/week	0.06–1	30	10–50
Fats	5/week	3–17.5	6	5–10

* IQR—interquartile range from 25th to 75th percentile.

## Supplementary Materials

The following are available online at http://www.mdpi.com/1010-660X/54/1/7/s1. Table S1. Consumption of food groups in relation with mother’s education in the infant’s age group before 5.9 months; Table S2. Consumption of food groups in relation with mother’s education in the infant’s age group above 6 months).
