# Supplementary material for: Eating Patterns and Food Choices of Latvian Infants during Their First Year of Life
Source: Medicina (Kaunas). 2018 Mar 23;54(1):7. doi: 10.3390/medicina54010007 (PMC6037245; doi:10.3390/medicina54010007)
Supplement: Supplementary file 1 [file medicina-54-00007-s001.pdf]

Table S1.Consumption of food groups in relation with mother's education in the infant's age group before 5.9 months.

Food group	University degree		Secondary education		Primary education or none		P for trend
	Consumption	CI95%	Consumption	CI95%	Consumption	CI95%	
**Vegetables**	13% (n=8)	7% - 23%	7% (n=3)	2% - 19%	7% (n=1)	1% - 30%	0.341
**Potatoes**	6% (n=4)	2% - 15%	10% (n=4)	4% - 22%	7% (n=1)	1% - 30%	0.767
**Fruits and berries**	6% (n=4)	2% - 15%	5% (n=2)	1% - 16%	7% (n=1)	1% - 30%	0.912
**Grains**	3% (n=2)	1% - 11%	5% (n=2)	1% - 16%	13% (n=2)	4% - 38%	0.153
**Meat**	2% (n=1)	0% - 8%	2% (n=1)	0% - 12%	7% (n=1)	1% - 30%	0.318
**Fish**	0% (n=0)	0% - 6%	0% (n=0)	0% - 8%	7% (n=1)	1% - 30%	0.046
**Cow's milk**	0% (n=0)	0% - 6%	5% (n=2)	1% - 16%	7% (n=3)	7% - 45%	0.067
**Dairy**	3% (n=2)	1% - 11%	0% (n=0)	0% - 8%	7% (n=1)	1% - 30%	0.868
**Eggs**	0% (n=0)	0% - 6%	0% (n=0)	0% - 8%	0% (n=0)	0% - 20%	0.151
**Legumes**	0% (n=0)	0% - 6%	0% (n=0)	0% - 8%	7% (n=1)	1% - 30%	0.046
**Fats**	5% (n=3)	2% - 13%	0% (n=0)	0% - 8%	7% (n=1)	1% - 30%	0.772

Table S2. Consumption of food groups in relation with mother's education in the infant's age group above 6 months.

**Food group**	**University degree**		**Secondary education**		**Primary education or none**		**P for trend**
	Consumption	CI95%	Consumption	CI95%	Consumption	CI95%	
**Vegetables**	90% (n=77)	81% - 94%	84% (n=43)	72% - 92%	67% (n=6)	35% - 88%	0.074
**Potatoes**	86% (n=74)	77% - 91%	84% (n=43)	72% - 92%	78% (n=7)	45% - 94%	0.545
**Fruits and berries**	79% (n=68)	69% - 86%	82% (n=42)	70% - 90%	89% (n=8)	57% - 98%	0.443
**Grains**	88% (n=76)	80% - 94%	88% (n=45)	77% - 94%	78% (n=7)	45% - 94%	0.539
**Meat**	72% (n=62)	62% - 80%	75% (n=38)	61% - 84%	78% (n=7)	45% - 94%	0.662
**Fish**	37% (n=32)	28% - 48%	31% (n=16)	20% - 45%	56% (n=5)	27% - 81%	0.789
**Cow's milk**	62% (n=53)	51% - 71%	71% (n=36)	57% - 81%	78% (n=7)	45% - 94%	0.187
**Dairy**	76% (n=65)	66% - 83%	80% (n=41)	68% - 89%	89% (n=8)	57% - 98%	0.308
**Eggs**	41% (n=35)	31% - 51%	47% (n=24)	34% - 60%	67% (n=6)	35% - 88%	0.737
**Legumes**	30% (n=26)	22% - 41%	28% (n=14)	17% - 41%	11% (n=1)	2% - 44%	0.310
**Fats**	73% (n=63)	63% - 81%	75% (n=38)	61% - 84%	67% (n=6)	35% - 88%	0.862
